# Short-term memory conjunctive binding in subjective cognitive decline: A PET biomarker-based study

**DOI:** 10.1007/s00415-026-13640-4

**Published:** 2026-01-30

**Authors:** M. A. Cecchini, A. Studart-Neto, N. C. Moraes, C. G. Carneiro, A. C. Gomes, C. A. Buchpiguel, S. M. D. Brucki, A. M. Coutinho, R. Nitrini, M. S. Yassuda

**Affiliations:** 1 Human Cognitive Neuroscience, Psychology, University of Edinburgh, Edinburgh, UK; 2 Department of Neurology, Faculdade de Medicina, Universidade de São Paulo (FMUSP), São Paulo, Brazil; 3 Laboratory and Division of Nuclear Medicine (LIM-43), Instituto de Radiologia, Hospital das Clínicas, Faculdade de Medicina, Universidade de São Paulo (HC-FMUSP), São Paulo, Brazil; 4 Gerontology, School of Arts, Sciences and Humanities, University of São Paulo, São Paulo, Brazil

**Keywords:** Memory binding, Conjunctive binding, Subjective cognitive decline, Cognitive marker, Alzheimer’s disease, Biomarkers

## Abstract

The Short-Term Memory Conjunctive Binding (STMCB) test assesses the ability to maintain integrated shape-colour associations in memory. It has been applied to detect Alzheimer’s disease (AD) across the continuum, from preclinical stages and subjective cognitive decline (SCD) to dementia. The objective of the present study was to examine whether the STMCB test can differentiate individuals at very early stages of AD from controls. The sample included 67 participants with normal performance on standard neuropsychological tests. Participants were classified as controls or as having SCD based on self-reported memory complaints. Twenty-three controls and 44 individuals with SCD completed the STMCB test. All individuals also underwent a comprehensive neuropsychological evaluation, amyloid ([^11^C]PIB) and FDG-PET scans. No significant group differences were observed in STMCB test performance between the groups. Furthermore, the STMCB test did not distinguish between amyloid-negative controls and SCD participants with amyloid pathology. These findings suggest that binding deficits may emerge later in the AD continuum, particularly when tau deposition or neurodegeneration is present.

**Supplementary Information:**

The online version contains supplementary material available at 10.1007/s00415-026-13640-4.

## Introduction

Conjunctive memory binding is the ability to hold in memory features (e.g., shapes and colours) integrated as a unique object. For instance, to recognise an object, such as our personal computer, we remember it as a whole, not as a combination between shapes, colours, textures, and size as separate entities. The short-term memory conjunctive binding (STMCB) test assesses this ability, focussing on integrating shapes and colours, and it has been used to detect Alzheimer’s disease (AD) patients across the AD continuum, from preclinical and subjective cognitive decline (SCD) to dementia stages (for a review, see [1]).

The STMCB test was able to identify AD [2, 3], mild cognitive impairment (MCI) [4–6], and patients at the preclinical stage of the disease, such as asymptomatic carriers of the E280A presenilin-1 mutation [3, 7], when compared to controls. In addition, the STMCB was able to discriminate AD from other types of dementia [8, 9], with the advantage of not being impacted by age [10, 11] or education [10]. The relevance of the STMCB test for clinical assessment of AD patients is being considered especially for very early stages of the disease, whilst the off-the-shelf neuropsychological tests would be more sensitive to detect MCI and dementia stages [12–14].

Regarding recent evidence on biomarkers, the AD continuum starts with amyloid deposition, followed by tau phosphorylation, leading to neurodegeneration [15, 16]. Thus, the STMCB test may identify AD patients before neurodegeneration starts (amyloid or amyloid + tau phases). A few studies have been done to understand the relationship between the STMCB test and biomarkers [7, 14, 17, 18]. Norton et al. [7] showed that the STMCB test correlated with amyloid-beta deposition using positron emission tomography (PET) of the brain with a Pittsburgh compound-B ([^11^C]PIB–PIB-PET) (*r*^2^ = 0.25, *p* = 0.03) in a sample with asymptomatic carriers of the PSEN1 E280A mutation. Cecchini et al. [17] compared controls and a group with amyloidosis without overt neurodegeneration, and the STMCB test was the only test that could differentiate these groups. Parra et al. [14] stratified a control sample based on a “binding cost” variable, defined as the percentage drop in performance between shape-only and shape-colour binding tasks. Participants who exhibited a steeper decline showed greater amyloid deposition in the parietal–occipital–temporal regions and fusiform gyrus.

Huyghe et al. [18] found that performance on a shape-colour binding task had a stronger association with tau deposition in the entorhinal cortex (*β* = − 6.594) compared to the shape-only condition (*β* = − 1.497). Interestingly, they found similar binding deficits in amyloid-positive cognitively unimpaired individuals and in MCI patients, suggesting that conjunctive binding impairments emerge early in the disease course. Furthermore, these impairments may plateau or decelerate as the disease progresses, a hypothesis also proposed by Cecchini and Della Sala [19] and Cecchini et al. [1].

To the best of our knowledge, only two studies have compared cognitively unimpaired individuals with those with SCD in samples of older adults [5, 12]. In these studies, SCD had significantly poorer performance on the binding task, despite performing at the normal range in other neuropsychological tests. However, these studies lacked biomarkers. Therefore, it was not possible to ascertain which participants had AD pathology, and it is still not clear how the STMCB test could contribute to detect sporadic AD patients at very early stages of the disease. Then, the objectives of the present study were (1) to verify whether the STMCB test could differentiate SCD patients from controls; and (2) to verify whether the STMCB test could differentiate patients at very early stages of AD, namely SCD patients who had significant amyloid deposition according to PIB-PET exams, from participants without AD pathology (SCD A− and controls A−).

## Methods

### Participants

An a priori power analyses were carried out using the G*Power 3.1.9.7 programme [20]. Assuming a power of 0.80 to detect a large effect size (*d* = 1.10) with *α* = 0.05 in a t test model, a minimum sample of 28 participants was required. The effect size used in the power analysis was based on a meta-analysis comparing the performance of controls and preclinical AD patients [1].

For this study, 185 older adults were invited to participate (age ≥ 60; education ≥ 4 years of formal schooling). Participants were recruited from three sites: (1) the outpatient clinic for older adults without dementia at the Geriatrics Outpatient Service of the Clinical Hospital, Faculty of Medicine, University of São Paulo; (2) the Open University of the Third Age programme at the University of São Paulo; and (3) a centre for the promotion of healthy ageing. These sites were selected, because they primarily serve cognitively unimpaired older adults. In addition, participants were recruited from the community through social media posts and newspaper coverage.

All participants underwent a clinical evaluation by a neurologist and a neuropsychologist. Exclusion criteria included a diagnosis of mild cognitive impairment (MCI) or dementia, the presence of psychiatric or neurological disorders, use of psychoactive medication, systemic or medical conditions that could affect cognition, and visual, auditory, or motor impairments that could interfere with neuropsychological testing.

The initial assessment with the neurologist included the following instruments: Mini-Mental State Exam (MMSE [21, 22]), Clinical Dementia Rating Scale (CDR [23, 24]), Montreal Cognitive Assessment (MoCA [25, 26]), Brief Cognitive Screening Battery (BCSB [27]), Geriatric Depression Scale (GDS [28, 29]), and Pfeffer Functional Activities Questionnaire (PFAQ [30, 31]). Following this assessment, 12 participants were excluded due to dementia diagnosis, one due to major depressive disorder, two due to recent cancer diagnoses, and two dropped out of the study.

In a second session, participants underwent a comprehensive neuropsychological testing, which included the following instruments: the Rey Auditory-Verbal Learning Test (RAVLT [32]), Logical Memory subtest from the Wechsler Memory Scale third edition (WMS-III [33, 34]), Rey–Osterrieth Complex Figure Test (ROCFT [35–37]), Forward and Backward Digit Span subtest from the Wechsler Adult Intelligent Scale third edition (WAIS-III [38, 39]), Trail Making Test parts A and B (TMT [40]), Verbal Fluency Tests (fluency with the letter “P” and semantic fluency with “animals”), and Boston Naming Test (BNT [41, 42]). Vocabulary and Matrix Reasoning subtests from the WAIS-III [38, 39] were used to estimate the intelligence quotient. In addition, the binding task was also administered. For this study, the performance on the following tests was included in the analysis: MMSE, MoCA, GDS, fluency tests, and RAVLT Total and Delayed scores.

Ninety-six participants were excluded from the present analyses due to a diagnosis of MCI, defined as performance at least 1.5 standard deviation below the mean in at least one test [43, 44]. Binding was not used for MCI or dementia diagnosis. In addition, five participants were excluded for not completing the binding test.

Participants were categorised as either controls or as having SCD based on their response to the question: “do you feel your memory is getting worse?” (following Jessen et al. [45]). Participants who answered “yes” were classified as SCD. The final sample comprised 44 individuals in the SCD group and 23 in the control group. All participants read and signed the informed consent form which was approved by the Ethics Committee from the University of Sao Paulo (reference number 62047616.0.0000.0068).

### Short-term memory binding test

The conjunctive memory binding test used in this study was the same as that employed in the previous research [2, 3, 17] and is publicly available at: https://www.strath.ac.uk/research/subjects/psychology/cognition/appliedcognitionlab/visualshort-termmemorybindingtestvSTMCBt. Prior to the main task, all participants completed a perceptual control task designed to assess their ability to perceive bound features (see Fig. SM1 in Online Resource 1, supplementary material). In this task, participants were asked to determine whether the items presented above a horizontal line were identical to or different from the items displayed below the line. All participants scored 14 or higher (out of a possible 16), indicating no perceptual impairments that could affect performance on the binding task.

**Fig. 1:**
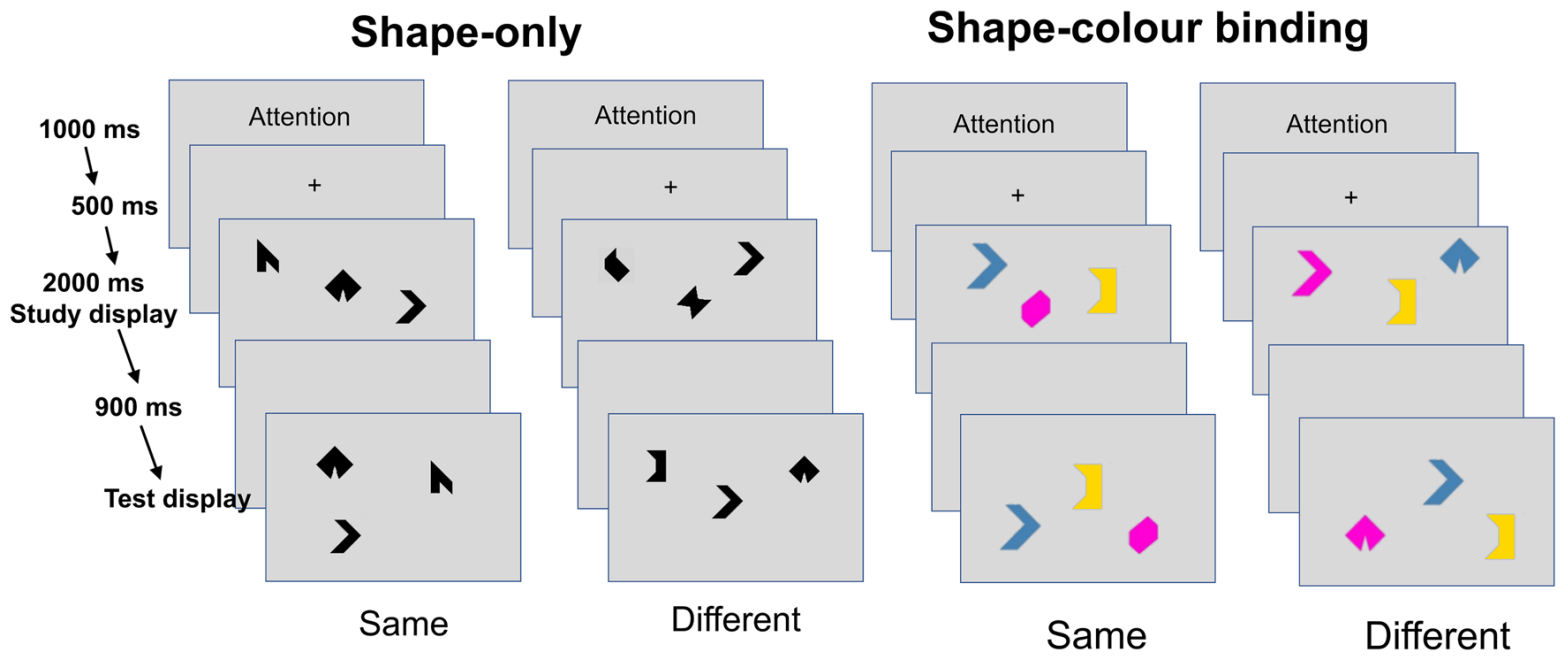
A representation of the short-term memory binding test and its two conditions: shape-only and shape-colour binding

The memory binding test involved the presentation of a study screen containing three items, followed by a 1-s interval and then a test screen, also with three items. Participants were required to indicate whether the items on the test screen were identical to those on the study screen or whether they differed. The test comprised 16 trials in total: 8 trials in which two items changed and 8 trials in which all items remained the same. Participants completed two conditions: (1) a shape-only condition, in which the items consisted of black, unnameable shapes (polygons); and (2) a shape-colour binding condition, in which the items were coloured polygons, and participants were instructed to detect changes in the specific combinations of shapes and colours. The two conditions were presented in a counterbalanced order, with half of the participants completing the shape-only condition first and the other half completing the shape-colour binding condition first. This procedure was used to control for potential order effects. Notably, item location was not relevant for the task, as the positions of the items changed between the study and test screens. Thus, participants were instructed to focus exclusively on the shapes or shape-colour pairings (Fig. 1).

Three variables were used in the analyses: the percentage of correct responses in the shape-only condition, the percentage of correct responses in the shape-colour binding condition, and the binding cost. Binding cost was calculated using the formula: Binding cost = 100 × (1 − [shape-colour binding/shape-only]). Alternative scoring methods commonly used in recognition tests (A’, d’, and hit rate minus false alarms) were also computed. However, as these yielded results consistent with the percentage-based measures, only the percentage scores are reported in the main text. The results of the alternative scoring methods are provided in supplementary material (Online Resource 1).

PET data acquisition and images classification (quantitative indices of AB burden and neurodegeneration).

All individuals were submitted to carbon-11-labelled Pittsburgh compound-B ([^11^C]PIB) and [^18^F]Fluorodeoxyglucose (FDG) positron emission tomography exams to detect amyloid deposition (PIB-PET) and access the neurodegeneration status (FDG-PET). Images were carried out at the Nuclear Medicine Center, Institute of Radiology, Hospital das Clínicas, Faculdade de Medicina, Universidade de São Paulo (CMN-InRad, HC-FMUSP) on a 3.0-Tesla SIGNA PET/MRI hybrid scanner (GE Healthcare, Milwaukee, WI). The average acquisition interval between the FDG and PIB-PET images was 18.5 (± 17.9) days. An on-site GMP cyclotron facility (PETtrace-880, GE-Healthcare) of the CMN-InRad (CInRad) was used, and the details regarding radiochemical production and image acquisition procedures were described previously [46, 47].

The participants were instructed to fast for at least 6 h before the FDG-PET exam. Capillary glucose was measured prior to the FDG-PET and should be lower than 180 mg/dL. The FDG was injected in a bolus (208.0 ± 17.9 MBq), and participants remained at rest, with eyes open and no audiovisual stimuli, for 30 min before imaging acquisition. PET-PIB images were acquired during 70 min with a dynamic protocol started immediately after intravenous administration of PIB (378.2 ± 37.5 MBq). In this work, the late-phase images were analysed, with an acquisition time between 50 and 70 min post-injection, reconstructed and condensed in time, creating single static images.

After the acquisition, FDG and PIB-PET images were reconstructed using an interactive reconstruction protocol (OSEM, 28 subgroups and 04 interactions in PET FDG and 28 subgroups and 02 interactions in PET-PIB), a standardised processing algorithm (VUE Point FX), smoothed using a cut-off value of 3.0 mm. Data were corrected for scattering, attenuation, and radioactive decay.

The standardised uptake value ratios (SUVRs) of the [11C]PIB uptake were calculated for 36 regions-of-interest (ROIs), generated using the Hammers N30R83 [48] map, placed on: prefrontal, orbitofrontal, parietal, temporal, occipital, parahippocampal, hippocampus, insula, amygdala, thalamus, basal ganglia, anterior cingulate and posterior cingulate cortices, and precuneus. The whole cerebellar tracer uptake was used to normalise the SUVR [49] using the PMOD™ software (PMOD Technologies LLC, Zürich, Switzerland). A composite ROI (metaROI) including all regions was used to generate the SUVr values for each individual [50].

Finally, to classify participants regarding the amyloid status (A+, A−), or core 1 biological AD diagnostic [16] visual inspection of PIB-PETs were carried out. A participant was considered as A+ if there was an increase in uptake in cortical grey matter (GM) areas causing a loss of GM to white matter (WM) contrast. This was aided by the 3D-SSP semi-quantitative method designed for the clinical analysis of brain PET amyloid imaging (Cortex ID Suite software, GE healthcare). The neurodegeneration status (N) was accessed qualitatively by the visual inspection of the FDG-PET images, performed by two board-certified nuclear medicine physicians, with support of the same 3D-SSP semi-quantitative method. Such qualitative analysis was described in detail in Coutinho et al. [46].

### Statistical analyses

The binding variables were normally distributed; however, Levene’s test indicated unequal variances in the shape-colour binding condition and the binding cost variable when comparing the control and SCD groups. Similarly, unequal variances were observed in the shape-colour condition when comparing A− controls and A+ SCD participants. Therefore, the Welch’s *t* test was used for these comparisons, as it is robust to violations of the homogeneity of variance assumption [51–53]. Effect sizes for group comparisons were calculated using Hedges’ *g*, which is more appropriate than Cohen’s *d* when dealing with unequal and small sample sizes [54]. Analysis of variance (ANOVA) was conducted to compare controls negative for amyloid and SCD groups split by the amyloid status (controls A−, SCD A−, SCD A+). We used the Bonferroni’s correction for multiple comparisons and described the Cohen’s *d* effect size for the comparison between control A− and both SCD groups (A− and A+). We did not include controls A + due to small sample (*n* = 5).

The proportion of neurodegeneration presence was compared across groups using Fisher’s exact test. The statistical analyses were performed using JASP v.0.95.4 and JAMOVI v.2.7.15, with the significance level set at *α* = 0.05.

## Results

The sociodemographic characteristics and cognitive performance across controls and SCD groups are presented in Table 1.

**Table 1:** Sociodemographic characteristics and cognitive performance comparing controls and SCD

	Controls (*n* = 23)	SCD (*n* = 44)	*p* value	Effect size
Age	76.52 (9.74)	73.39 (7.24)	0.182	0.361
Education	13.78 (5.56)	14.02 (3.82)	0.854	0.050
MMSE	28.87 (1.14)	28.91 (0.88)	0.886	0.038
MoCA	24.78 (2.28)	24.36 (2.2)	0.473	0.185
GDS	1.09 (1.24)	2.2 (1.46)	0.002	0.817
SVF animals	19.09 (4.64)	17.23 (5.74)	0.158	0.352
PVF (p)	14.13 (5.18)	14.61 (4.77)	0.712	0.096
RAVLT total	44.22 (7.82)	40.93 (8.43)	0.118	0.400
RAVLT delayed	8.04 (2.21)	7.55 (2.44)	0.402	0.211
Shape-only	13.04 (2.16)	13.41 (1.19)	0.428	0.222
Shape-colour binding	11.52 (2.23)	11.25 (1.82)	0.618	0.132
Binding cost	8.80 (25.21)	15.53 (15.39)	0.239	0.327
PIB-PET SUVr	1.31 (0.25)	1.38 (0.44)	0.441	0.184
FDG-PET (N+ AD/N+ non-AD/N−)	0/4/19	1/7/36	1.000	–

*SCD* subjective cognitive decline, *N* neurodegeneration measured with FDG-PET; signals refers to negative (−) or positive (+) for the biomarker presence; *N+ AD* abnormal FDG-PET with a metabolic compatible with Alzheimer’s disease pattern, *N+ non-AD* abnormal FDG-PET not compatible not suggestive or atypical for AD, *MMSE* Mini-Mental State Exam, *MoCA* Montreal Cognitive Assessment, *GDS* Geriatric Depression Scale, *SUVr* standardised uptake value ratios for amyloid-beta, *SVF* semantic verbal fluency, *PVF* phonemic verbal fluency, *RAVLT* Rey Auditory-Verbal Learning Test, *STMCB* Short-Term Memory Binding

*p* values were obtained using *t* tests, with the exception of the FDG-PET variable, which was compared using Fisher’s exact test; effect sizes were estimated using the Hedges’ g formula

Groups were similar in age and education, biomarker profiles for amyloid and neurodegeneration, differing only in the GDS score, with higher scores for the SCD group. The sociodemographic and cognitive characteristics of the sample, divided into amyloid-negative controls and amyloid-positive SCD participants, are presented in Table 2.

**Table 2:** Sociodemographic characteristics, cognitive performance, and FDG-PET (N) status comparing controls and patients with subjective cognitive decline with amyloidosis

	Controls A− (*n* = 17)	SCD A− (*n* = 31)	SCD A+ (*n* = 12)	*p* value	EF controls X SCD A−	EF controls X SCD A+	EF SCD A− X SCD A+
Age	75.47 (9.39)	71.55 (6.89)	77.75 (6.58)	0.042	0.515	0.299	0.814
Education	14.35 (5.49)	13.74 (3.81)	14.58 (4.06)	0.816	0.139	0.052	0.192
MMSE	29.00 (1.17)	28.81 (0.91)	29.08 (0.79)	0.647	0.199	0.086	0.285
MoCA	25.18 (2.27)	24.58 (1.86)	23.75 (2.99)	0.247	0.267	0.639	0.372
GDS	1.00 (1.17)^bc^	2.13 (1.36)^a^	2.42 (1.78)^a^	0.013	0.804	1.009	0.205
SVF (animals)	19.53 (5.13)	17.94 (6.34)	15.58 (3.78)	0.183	0.285	0.706	0.421
PVF (p)	14.06 (5.77)	15.00 (4.71)	13.33 (5.02)	0.599	0.185	0.143	0.328
RAVLT total	45.88 (8.04)	41.42 (8.58)	40.50 (8.19)	0.145	0.534	0.644	0.110
RAVLT delayed	8.41 (2.29)	7.84 (2.54)	7.00 (2.13)	0.304	0.239	0.588	0.349
Shape-only	13.53 (2.04)	13.39 (1.17)	13.58 (1.17)	0.906	0.097	0.037	0.134
Shape-colour binding	11.35 (2.34)	11.19 (1.83)	11.42 (1.93)	0.935	0.079	0.032	0.111
Binding cost	14.06 (22.24)	15.80 (15.88)	15.46 (15.34)	0.948	0.098	0.079	0.019
PIB-PET SUVr	1.20 (0.11)^c^	1.17 (0.09)^c^	1.92 (0.53)^ab^	< 0.001	0.145	2.883	3.028
FDG-PET (N+ AD/N+ non-AD/N−)	0/2/15	1/3/26	0/3/9	0.532	–	–	–

*SCD* subjective cognitive decline, *A* amyloid deposition measured with [11C]PIB-PET, *N* neurodegeneration measured with FDG-PET, signals refers to negative (−) or positive (+) for the biomarker presence, *N+ AD* abnormal FDG-PET with a metabolic compatible with Alzheimer’s disease pattern, *N+ non-AD* abnormal FDG-PET not compatible not suggestive or atypical for AD, *MMSE* Mini-Mental State Exam, *MoCA* Montreal Cognitive Assessment, *GDS* Geriatric Depression Scale, *SUVr* standardised uptake value ratios for amyloid-beta, *SVF* semantic verbal fluency, *PVF* phonemic verbal fluency, *RAVLT* Rey Auditory Verbal Learning Test, *STMCB* Short-Term Memory Binding

*p* values were obtained using ANOVA tests; proportions of neurodegeneration participants across groups were obtained using Fisher’s exact test; effect sizes were estimated using the Cohen’s *d* formula

^a^Differed from controls A−

^b^Differed from SCD A−

^c^Differed from SCD A+

The groups were comparable in terms of age and educational level. However, they differed significantly on the GDS, with participants in the SCD group presenting higher GDS scores. No statistically significant differences were found between the groups in any of the memory binding measures, nor in FDG-PET biomarker parameters.

## Discussion

This study aimed to verify whether the memory binding test could differentiate individuals with SCD from controls, and SCD with biological evidence of AD (A+) from controls without AD (A−). Negative results were found in both comparisons, with groups showing nearly identical scores across all memory binding test variables. Interestingly, and contrary to our expectations, effect sizes for all binding tasks decreased when the sample was stratified by amyloid status. That is, when we ascertained that participants without AD pathology (controls A−) were compared with preclinical AD (SCD A+), their performance was essentially the same on the binding tasks. In addition, despite being older (*d* = 0.814), the SCD A+ group showed slightly better performance on the shape-colour binding task compared with the SCD A− group (*d* = 0.111). These findings contrast with the previous studies reporting that memory binding tasks could differentiate controls from SCD [5, 12] or preclinical AD [3, 7, 18, 55].

Several hypotheses may account for the present findings. First, the memory binding test may lack sensitivity to detect cognitive changes when only amyloid pathology is present. In this study, we were unable to assess tau status, as neither tau-PET imaging nor cerebrospinal fluid (CSF) analyses were available. We could, however, access neurodegeneration (“N” status), and showed that most of the individuals included in both groups had normal FDG-PET scans (N−), indicating that the SCD A+ individuals in the sample were in very early stages of the AD continuum (A+N−).

Additionally, the N+ individuals had predominantly a non-AD pattern, most of them with a temporal/limbic pattern. Nonetheless, even if the memory binding test is not sensitive enough to detect individuals with isolated amyloid pathology, identifying deficits at the A+T+ stage, or A+N+ if tau tests are unavailable, remains clinically relevant. A growing body of research suggests that tau pathology is more closely associated with cognitive impairment [56], cognitive decline [57], functional decline [58], and increased risk for progression to MCI or dementia [59, 60] than amyloid burden alone. For instance, Ossenkoppele et al. [60] found that individuals with A+T+ have been shown to have a six-to-sevenfold increased risk of progressing to MCI within 3–5 years, compared to those with A+T− profiles. Additionally, after stablishing the amyloid presence (Stage A), the biological staging of AD is provided by tau-PET (Stages B-D [16]). Therefore, adding tau-PET in future studies would provide refinement to when, or at which biological stage, binding deficits occurs.

There is mixed evidence regarding the relationship between memory binding deficits and tau pathology. Huyghe et al. [18] found that tau accumulation in the entorhinal cortex, but not in the inferior temporal lobe, was predictive of conjunctive binding deficits in cognitively unimpaired individuals. Therefore, both the presence and the regional distribution of tau pathology (i.e., not just T+/T− status, but specific deposition sites) may be critical for understanding memory binding deficits in the AD continuum (see [61]). Norton et al. [7], on the other hand, reported no significant association between performance on the conjunctive memory binding test and tau deposition in the entorhinal cortex, although a significant correlation was found with general amyloid burden.

It is possible to reconcile these apparently contradictory findings. If the participants in Huyghe et al. [18] had tau deposition extending beyond the entorhinal and perirhinal cortices, they may have been at a more advanced disease stage than those in Norton et al. [7]. Tau pathology is known to accumulate first in the entorhinal and perirhinal cortices (Braak Stages I–II) and only later in the inferior temporal lobe [62]. Thus, an association between binding performance and entorhinal tau may only emerge once tau has already spread to additional regions, indicating a more biologically advanced stage of AD. However, this interpretation remains speculative, because the samples are not directly comparable: Norton et al. [7] examined individuals with genetic AD, whereas Huyghe et al. [18] studied a sporadic AD cohort. Consequently, the precise point along the AD continuum at which conjunctive binding deficits first appear is still uncertain.

Of note, several factors may interact with cognition and its progression along the AD continuum, including age, apolipoprotein E (APOE) ε4 status, and potentially cognitive reserve [63–67]. Mufson et al. [65] reported that the combination of Braak stage, age, and APOE status, rather than Braak or APOE alone, was significantly associated with trajectories of cognitive decline. Pettigrew et al. [66] found that higher cognitive reserve may mitigate APOE-related cognitive deterioration, whilst Groot et al. [63] and van Loenhoud et al. [67] showed that the influence of cognitive reserve on cognitive performance is particularly relevant in predementia stages. Our SCD sample was relatively highly educated, with an average of approximately 14 years of formal schooling. Although binding deficits have been reported in SCD participants with an average of around 16 years of education [12], it is possible that cognitive reserve mitigated binding deficits in our SCD groups. Therefore, future research should aim to characterise cognitively unimpaired individuals more comprehensively by incorporating biomarkers of amyloid, tau, and neurodegeneration, whilst controlling for age, APOE status, and cognitive reserve. Such an approach will help clarify the temporal and pathological onset of binding impairments.

Another hypothesis to explain our findings is that the memory complaints reported by the SCD group may have been driven by depressive symptoms. Although all participants scored below the clinical threshold of 5 on the GDS and did not meet criteria for major depression, the GDS significantly differentiated the groups, with a large effect size. This suggests that subclinical depressive symptoms may be associated with the cognitive complaints observed in the SCD group.

The association between subjective memory and depressive symptoms was previously identified [68–71]. Psychological characteristics, such as depression, anxiety, stress, or personality, were better to predict subjective memory when compared to objective memory tests in both cross-sectional [70, 72–80] and longitudinal [68, 69, 71, 81–84] studies. These findings point to the possible lack of validity of subjective measures of memory (see Uttl and Kibreab [85]). That is, subjective and objective memory may represent different and uncorrelated constructs. Crumley et al. [86] illustrated this in a meta-analysis, in which they found a negligible correlation of 0.06 between them. Therefore, objective cognitive measures and biomarkers may be a more reliable way of assessing patients early in the AD continuum when compared with subjective memory.

Differences in the binding task format may have also contributed to the contrasting results with the previous studies. In the present study, a version with 16 trials per condition was used, whereas earlier studies typically employed a version with 32 trials [3, 5, 7, 12, 18]. Although the 16-trial version has demonstrated the ability to detect MCI [4], AD [4, 17], and marginally discriminated SCD in Forno et al. [12] (*p* = 0.052, effect size = 0.467), the reduced number of trials may have compromised the test’s sensitivity to detect subtle deficits, such as those present in the A+T−N− stage of AD.

Additionally, the task used in this study included three items per trial. The optimal memory load for detecting early AD remains unclear, with mixed findings in the literature. Using three items per trial has been suggested when dealing with preclinical AD cases [12, 55], but Huyghe et al. [18] reported better diagnostic performance with two items. Moreover, Huyghe et al. [18] found that two-item trials were more predictive of tau deposition in the entorhinal cortex. They also showed that using three items per trial increased the binding cost in control participants (from a mean of 4.86 with two items to 14.69 with three). The increased binding cost in controls using three items was also found in Parra et al. [6] and Valdés Hernández et al. [87], but not in other studies [2, 3, 5, 12, 55]. It is therefore possible that the higher memory load used in the present study may have reduced the accuracy of the test in detecting preclinical AD, by disproportionately affecting control participants’ performance in the binding condition.

The rationale for using different set sizes is to avoid overloading visual working memory capacity. Poor performance on binding tasks should reflect difficulties specifically in maintaining bindings, rather than a general working memory deficit. If the set size exceeds working memory capacity, test performance may be confounded by non-specific limitations, introducing noise into the measure. This may explain why smaller set sizes (e.g., two items per trial) proved more effective in discriminating MCI patients from controls [6]: the binding difficulty of MCI patients became more evident when the task demands did not surpass their general working memory capacity. To improve sensitivity, future studies should include binding tests with varying set sizes to detect individuals in preclinical stages of AD. Alternatively, as proposed by Cecchini et al. [1], tailoring task difficulty to each individual (rather than applying a fixed level across groups) may provide a more precise assessment.

Finally, longitudinal studies involving the memory binding task are necessary, particularly given that not all individuals at risk for AD will go on to develop cognitive impairment or dementia [88, 89]. If the binding task can identify individuals at risk or AD and predict which of them are likely to progress to MCI or dementia, this would provide strong evidence for its clinical utility.

The limitations of the study should be acknowledged. In addition to the absence of tau biomarker data, the overall sample size was small, particularly when the sample was stratified by amyloid status, which reduced the statistical power to detect group differences. However, it is worth noting that the effect sizes for all group comparisons on the binding tasks were negligible or very small, suggesting that the lack of significant findings is unlikely to be solely due to reduced power. In addition, the recruitment strategy and the conservative criteria used to classify participants with MCI may have limited the representativeness of our sample relative to the general population.

In sum, our results indicate that the memory binding test did not distinguish individuals with SCD or those with amyloid positivity from controls. Stratification by amyloid status further reduced effect sizes, challenging the sensitivity of the test to isolated amyloid pathology at the earliest stage of the Alzheimer’s continuum. Binding deficits may emerge only once tau is present and spread in cortical areas in addition to the entorhinal cortex. In that case, it remains to be determined whether binding deficits precede the episodic memory impairments typically observed in more biologically advanced stages of AD [16]. In addition, methodological choices, such as reduced trial number and a higher set size, may have attenuated sensitivity by increasing measurement noise.

Given these constraints, future studies should combine full biomarker characterisation, longitudinal follow-up, and adaptive task designs that vary set size and trial exposure. Until such evidence is available, memory binding test should be interpreted with caution for preclinical AD and considered only in conjunction with biomarkers, comprehensive neuropsychological assessments, and measures of mood to improve early diagnostic accuracy.

## Supplementary Information

Below is the link to the electronic supplementary material.

Supplementary file1 (PDF 145 KB)

## Data Availability

Given the limited availability of PET scans in Brazil, there is a heightened risk of participant identification in research when demographic information (e.g., sex, age, and education) is combined. Therefore, for ethical reasons, the dataset is not publicly available. Further analyses may be requested from the corresponding author.
