# Supplementary material for: Short-term memory conjunctive binding in subjective cognitive decline: A PET biomarker-based study
Source: J Neurol. 2026 Jan 30;273(2):107. doi: 10.1007/s00415-026-13640-4 (PMC12858614; doi:10.1007/s00415-026-13640-4)
Supplement: Supplementary file 1 — Supplementary file1 (PDF 145 KB) [file 415_2026_13640_MOESM1_ESM.pdf]

Supplementary Material

Short-term Memory Conjunctive Binding in Subjective Cognitive Decline - a PET biomarker-based study.

Journal of Neurology

Cecchini MA[1], Studart-Neto A, Moraes NC, Carneiro CG, Gomes AC, Buchpiguel CA, Brucki SMD, Coutinho AM, Nitrini R, Yassuda MS.

1. Human Cognitive Neuroscience, Psychology, University of Edinburgh, Edinburgh, United Kingdom. mario.cecchini@ed.ac.uk

Fig. SM1

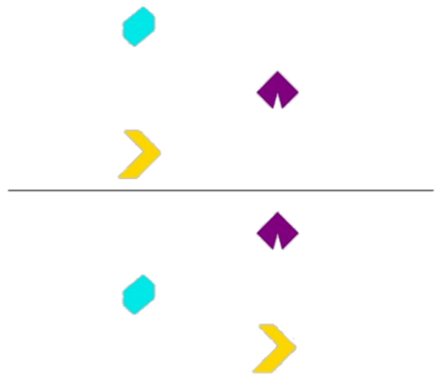

Fig. SM1. A representation of the perception task of the short-term memory binding test.

In the Perception task, participants should say if the combinations between shapes and colours was the same or if they are different when comparing the items above and below the line.

Results obtained using alternative measures from the short-term memory binding test

Table SM1. Performance comparing controls and subjective cognitive decline patients

	Controls (n=23)	SCD (n=44)	P-value	Effect size
Shape-only A’	3.61(2.33)	3.57(1.45)	0.946	0.020
Shape-only d’	1.92(0.83)	2.09(0.48)	0.377	0.268
Shape-only (Hits-FA)	0.636(0.27)	0.696(0.15)	0.322	0.304
Shape-colour Binding A’	0.788(0.13)	0.776(0.13)	0.713	0.094
Shape-colour Binding d’	1.31(0.79)	1.22(0.70)	0.625	0.125
Shape-colour Binding (Hits-FA)	0.440(0.26)	0.411(0.23)	0.638	0.120
Shape minus binding	1.52(3.34)	2.16(2.07)	0.394	0.254

Legend. SCD = subjective cognitive decline; p-values were obtained using t-tests; effect sizes were estimated using the Hedges’ g formula.

Table SM2. Performance comparing controls amyloid negative x SCD amyloid negative x SCD amyloid positive

	Controls A- (n=17)	SCD A+ (n=12)	P-value	Effect size
Shape-only A’	4.11(2.36)	3.34(1.19)	0.260	0.400
Shape-only d’	2.11(0.78)	2.02(0.46)	0.711	0.131
Shape-only (Hits-FA)	0.700(0.25)	0.677(0.14)	0.766	0.105
Shape-colour Binding A’	0.783(0.15)	0.798(0.12)	0.769	0.107
Shape-colour Binding d’	1.318(0.88)	1.368(0.81)	0.877	0.057
Shape-colour Binding (Hits-FA)	0.441(0.29)	0.458(0.27)	0.873	0.059
Shape minus shape-colour binding	2.18(3.26)	2.17(2.12)	0.992	0.003

Legend: SCD = subjective cognitive decline; A = Amyloid deposition measured with [11C]PIB-PET; signals refers to negative (-) or positive (+) for the biomarker presence; p-values were obtained using ANOVAs; effect sizes were estimated using the Hedges’ g formula.
